# Topochemical conversion of a dense metal–organic framework from a crystalline insulator to an amorphous semiconductor†
†Electronic supplementary information (ESI) available: PXRD, impedance, TGA, IR, XPS, PDF, ESR, and CIF files. CCDC 1018776–1018778. For ESI and crystallographic data in CIF or other electronic format see DOI: 10.1039/c4sc03295k



**DOI:** 10.1039/c4sc03295k

**Published:** 2014-12-01

**Authors:** S. Tominaka, H. Hamoudi, T. Suga, T. D. Bennett, A. B. Cairns, A. K. Cheetham

**Affiliations:** a Department of Materials Science and Metallurgy , University of Cambridge , Charles Babbage Road , Cambridge CB3 0FS , UK . Email: akc30@cam.ac.uk ; Fax: +44 (0) 1223 334567 ; Tel: +44 (0) 1223 767061; b International Center for Materials Nanoarchitectonics (WPI-MANA) , National Institute for Materials Science (NIMS) , 1-1 Namiki , Ibaraki 305-0044 , Japan . Email: TOMINAKA.Satoshi@nims.go.jp ; Tel: +81 29 860 4594; c Waseda Institute for Advanced Study (WIAS) , Waseda University , 3-4-1 Okubo, Shinjuku , Tokyo 169-8555 , Japan; d Department of Chemistry , University of Oxford , Inorganic Chemistry Laboratory , South Parks Road , Oxford, OX1 3QR , UK

## Abstract

The topochemical conversion of a dense, insulating metal–organic framework (MOF) into a semiconducting amorphous MOF is described. Treatment of single crystals of copper(i) chloride trithiocyanurate, Cu^I^Cl(ttcH_3_) (ttcH_3_ = trithiocyanuric acid), **1**, in aqueous ammonia solution yields monoliths of amorphous Cu^I^_1.8_(ttc)_0.6_(ttcH_3_)_0.4_, **3**. The treatment changes the transparent orange crystals of **1** into shiny black monoliths of **3** with retention of morphology, and moreover increases the electrical conductivity from insulating to semiconducting (conductivity of **3** ranges from 4.2 × 10^–11^ S cm^–1^ at 20 °C to 7.6 × 10^–9^ S cm^–1^ at 140 °C; activation energy = 0.59 eV; optical band gap = 0.6 eV). The structure and properties of the amorphous conductor are fully characterized by AC impedance spectroscopy, X-ray photoelectron spectroscopy, X-ray pair distribution function analysis, infrared spectroscopy, diffuse reflectance spectroscopy, electron spin resonance spectroscopy, elemental analysis, thermogravimetric analysis, and theoretical calculations.

## Abstract 2

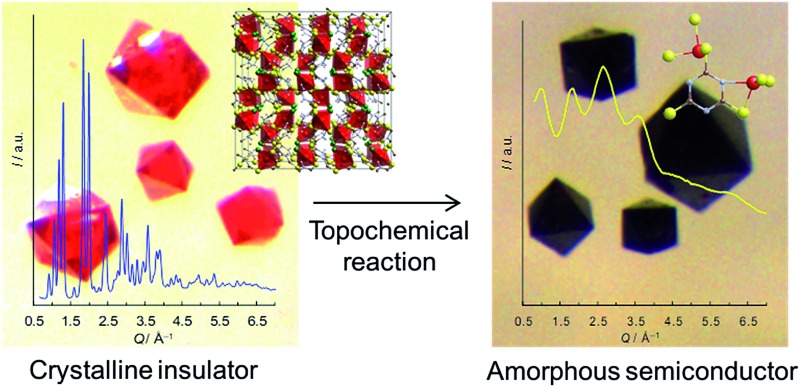
A dense, insulating metal–organic framework (MOF), is successfully converted into a semiconducting amorphous MOF *via* a topochemical route.

Solid-state materials for applications in electronic and optoelectronic devices consist almost entirely of classical inorganic and organic systems, *e.g.*, metals, oxides and polymers. Metal–organic frameworks (MOFs), on the other hand, represent a new class of solid-state materials comprising metal ions and organic ligands,^1^ which often combine the advantages of the former (*e.g.*, magnetism and optical properties) with those of the latter (*e.g.*, flexibility and lightness), making them interesting candidates for novel functional materials. The majority of MOFs studied to date are *nanoporous* and are being widely explored for applications in areas such as gas storage, gas separation and catalysis,^2^ though they are increasingly being investigated as promising materials for electronic and other functional devices.^3^ In a parallel development, *dense* MOFs, which are more analogous to classical solid state materials,^4^ are rapidly emerging as promising materials for applications in optoelectronics, ferroelectrics, multiferroics, batteries, magnets and other areas.^5,6^ The present work concerns an insulating, dense MOF that can be converted into an amorphous semiconducting phase by post synthetic modification.

Coordination polymers having electrical conductivity are mostly of low dimensionality,^7^ but the three-dimensional structures Cu–TCNQ and Ag–TCNQ (TCNQ = tetracyanoquinodimethane) are known to be highly conductive dense MOFs having electronic conductivities of ∼10^–2^ S cm^–1^ at 295 K.^8^ Porous MOFs having intrinsic electrical conductivity are extremely rare, though Cu[Cu(pdt)_2_] (pdt = 2,3-pyrazinedithiolato) shows conductivity of 6 × 10^–4^ S cm^–1^ at 300 K (1.0 × 10^–8^ S cm^–1^ for Cu[Ni(pdt)_2_]),^9,10^ and a tetrathiafulvalene-based porous MOF exhibits a high charge mobility of 0.2 cm^2^ V^–1^ s^–1^.^11^ Very recently, tunable electrical conductivity (10^–6^ to 7 S cm^–1^) in the porous MOF, [Cu_3_(btc)_2_] (btc = benzen-1,3,5-tricarboxylato) was achieved by the infiltration of TCNQ guest molecules.^12^

Coordination of transition metals with polarisable groups such as sulphur or cyano units is a well-established strategy for creating conductive coordination polymers.^7,14^ Trithiocyanuric acid (C_3_H_3_N_3_S_3_: ttcH_3_) has three thiol groups and triazine conjugation,^6^ and thus appears to be a good candidate for forming 3D conducting networks. Such networks (dimensionality, I^0^O^3^)^15^ of Cu^I^X(ttcH_3_) (X = Cl^–^, Br^–^ and I^–^) can be made,^13^ but unfortunately the structures are composed of isolated CuClS_3_ tetrahedra connected by neutral ttcH_3_ molecules in the thioketone form (Fig. 1). Electronic conductivity is not observed and the phases have optical band gaps of 1.8–2.3 eV (valence band of Cu orbitals and conduction band of S orbitals).^13^ In the expectation that the product would contain an inorganic Cu–S–Cu network, we have therefore explored the possibility of dehalogenating Cu^I^Cl(ttcH_3_) and turning the neutral ttcH_3_ molecules into anions to form Cu^I^(ttcH_2_). Dehalogenation can indeed be achieved, and moreover we discovered that the product forms an amorphous network that is a reasonable semiconductor.

**Fig. 1:**
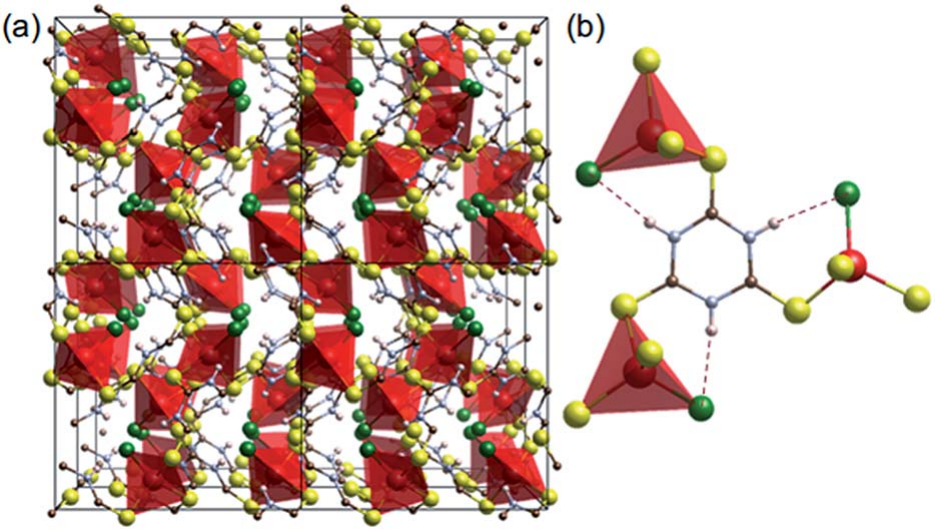
Crystal structure of [CuCl(ttcH_3_)].^13^ (a) 2 × 2 × 2 cells. (b) Connectivity of CuClS_3_ tetrahedral with the thioketone form of trithiocyanuric acid molecules (red: Cu, green: Cl, yellow: S, blue: N, brown: C, and white: H). Dotted lines show hydrogen bonding.

## Experimental

### Synthesis

The CuCl(ttcH_3_) crystals, **1**, were synthesized *via* a solvothermal route in which 0.50 mmol of CuCl (95%, Alrdich) and 0.5 mmol of ttcH_3_ (>98%, TCI) were heated overnight in 20 mL of acetonitrile (99.9%, Fisher) at 60 °C and then kept at 80 °C for 2 days. Reddish orange, octahedral crystals of 10–200 μm size were collected by vacuum filtration and rinsed with ethanol (yield = 95%). The chemical composition was determined by elemental analysis carried out at the Department of Chemistry, University of Cambridge: (found, calcd wt% for C_3_H_3_N_3_S_3_CuCl), C(13.33, 13.04), H(1.00, 1.09), N(15.26, 15.21) and Cl(12.96, 12.83).

Crystals of **1** were treated in 0.43% aqueous ammonia (pH = 11.4, 35% NH_3_ aq. + distilled water; both supplied by Fisher) at room temperature for two days. The colour of the crystals turned from orange to deep red with shape retention. The product was rinsed with distilled water, and the colour further darkened to black, compound **2**. The product was then dried at 130 °C under vacuum for >6 h, compound **3**. The chemical compositions were as follows: **2**, found C, 10.76; H, 1.23; N, 14.70; Cl, 0.00 wt% (in moles, C : H : N = 1.00 : 1.37 : 1.17). **3**, found C, 12.33; H, 0.45; N, 14.28; Cl, 0.00 wt% (in moles, C : H : N = 1.00 : 0.43 : 0.99). The reaction was judged to be completed after 2 days as the composition of a sample treated in aqueous ammonia for 5 days was the same as **3**: found C, 12.24; H, 0.50; N, 14.39; Cl, 0.00 wt% (in moles, C : H : N: = 1.00 : 0.49 : 1.01).

### Photoelectron spectroscopy

X-ray photoelectron spectra were collected on an Ulvac-Phi PHI Quantera SXM spectrometer with monochromatic Al Kα X-rays at 15 kV, pass energy of 55 eV, energy step of 0.1 eV, and dual beam charge neutralization (electron: 1.4 eV and 10 μA; Ar^+^: 7 eV and 30 nA). Narrow beams of 0.2 mm*φ* at 25 W and 0.05 mm*φ* at 12.5 W were used for compounds **1** and **3**, respectively. The photoelectron escape angle was 45°. The spectra were referenced to adventitious C 1s at a binding energy of 248.48 eV. The samples were fixed on conductive carbon tape. The spectra were corrected through background subtraction with the Shirley method using the COMPRO program.

### Pair distribution functions

X-ray total scattering data for obtaining high-resolution pair distribution functions (PDFs) were measured on the I15 beamline at the Diamond Light Source, U.K. The data were recorded on a Perkin Elmer flat panel 1621 EN detector at a beam energy of 72 keV (*λ* = 0.17220 Å). The samples were sealed in Kapton capillaries (inner diameter: 1.02 mm; outer diameter: 1.105 mm; Cole–Parmer). The 2D scattering data were converted into 1D patterns using the program Fit2D.^16^ Total scattering data were also collected with Ag Kα (*λ* = 0.560883 Å) operated at 50 kV, 40 mA for 22 h (RINT RAPID-S with a total-reflection collimator, Rigaku). The samples were sealed in Lindeman glass capillaries (inner diameter: 0.48 mm; outer diameter: 0.5 mm; Hilgenberg). Background subtraction, X-ray polarization correction, absorption correction, and Compton scattering correction were performed using the program PDFgetX2,^17^ and the structure functions (*Q*_max_ = 24.5 Å^–1^, spatial resolution of 0.256 Å for the synchrotron data) were converted into PDFs.

The PDFs were analyzed by curve fitting using the program PDFgui.^18^ In order to model chemical short-range order in **3**, which results in the sharp peaks in the PDF, (i) the ttc^3–^ anion was constrained to be a flat, regular hexagonal molecule having flexible N–C and S–C bond lengths without hydrogen atoms; (ii) two CuS_3_ coordination units were used as the initial model, as found in **1** (occupancy of Cu = 0.9); and (iii) the clusters described above were treated as isolated molecules. Weak broad oscillation underneath the sharp features is considered to reflect the arrangement of the clusters as well as the number density, and was simulated by moving the coordinates of the original Cu sites with larger atomic displacement parameters (with a spherical particle model).^19^ This model for the broad features is not so definitive as that for the sharp features, which is supported by other measurements, but is useful for simulating the broad features originating from the average number density. The isolated molecules to fit the sharp peaks and the Cu sites distribution to fit the broad oscillation were described as two different phases in the PDFgui program, and refined simultaneously. Structures were visualized using the program VESTA.^20^

### X-ray diffraction

The crystal structure of **1** was determined at 120 K, 303 K, and 400 K by single crystal diffractometry using an Oxford Diffraction Gemini A Ultra X-ray diffractometer operating with Cu Kα radiation. The structure was solved by direct methods and then refined by least squares methods using the SHELX program^18^ within the Olex2 interface.^19^ All non-hydrogen atoms were refined anisotropically and hydrogen atoms were refined isotropically. CIF files are available for the structures.†

The phase purity was confirmed by powder X-ray diffraction (PXRD). The PXRD data were collected using a Bruker-AXS D8 diffractometer with Cu Kα radiation in the Bragg–Brentano geometry. The patterns were analyzed by Pawley fitting using the GSAS-II software,^21^ and plotted with zero-shift correction and background subtraction. The lattice constants were refined by the Pawley fitting of the powder X-ray diffraction pattern (*Pa*3, *a* = 11.77198 (6) at RT, *R*_w_ = 9.11%, *R*_wb_ = 7.84, *S* = 1.08, Fig. S1 in ESI†).^22^

### Conductivity measurements

The conductivities were measured under a nitrogen atmosphere by the AC impedance method using a Gamry Interface1000 electrochemical instrument in the frequency range of 1 MHz to 10 mHz at an AC amplitude of 100 mV. For the powder measurements, the samples (100–130 mg) were gently ground using a pestle and mortar and then pelletized (1 cm*φ*, ∼0.6 mm thick) at 0.5 GPa for 5 min with a few drops of water; the pressure was gradually decreased over 1 h. The pellets were dried at 130 °C under vacuum overnight. The data were collected using a closed cell flushed with nitrogen. After equilibration at 140 °C for a day, impedance spectra were collected at 20 °C intervals on cooling from 140 °C to 20 °C with an equilibration time of >8 h. The current–voltage characteristics were measured by cyclic voltammetry at the scan rate of 10 mV s^–1^ at 25 °C using a Hokuto HZ5000 electrochemical system. Furthermore, conductivities of single crystals and single monoliths were measured by a single-crystal impedance method using microelectrodes.^6,23^ The samples were mounted on a quartz chip having microelectrodes with a 80 μm gap and the temperature was controlled with a thermoelectric module from 20 °C to 140 °C.^6^

### Other measurements

Thermogravimetric analysis and differential scanning calorimetry were performed simultaneously from room temperature to 600 °C using a TA Instruments Q600 SDT instrument with an air flow of 100 mL min^–1^ at a heating rate of 5 °C min^–1^. Fourier-transform infrared spectroscopy (FTIR) was carried out using a Bruker Tensor 27 infrared spectrometer with a diamond attenuated total reflectance (ATR) attachment. The data were collected in the wavenumber range from 520 to 4000 cm^–1^ at room temperature. Diffuse reflectance spectra were obtained using a PerkinElmer Lamda 750 spectrometer with an integrating sphere detector in the wavelength range from 2500 to 220 nm. The powder samples were diluted to 10 wt% with dry BaSO_4_. The diffuse reflectance spectra were converted into Kubelka–Munk (K–M) functions with the concentration corrections. Electron spin resonance (ESR) spectra were collected at room temperature using a JEOL JES-TE200 ESR spectrometer with a 100 kHz field modulation.

## Results and discussion

### Appearance & composition

**Fig. 2:**
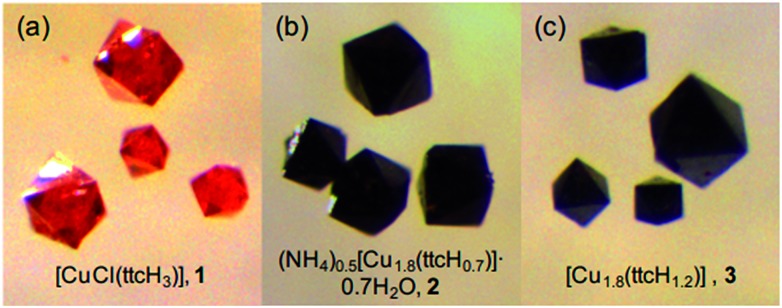
Photos of (a) **1** CuCl(ttcH_3_) crystals, (b) **2** – *i.e.* **1** treated in aqueous ammonia for 2 days, and (c) **3** – *i.e.* **2** dried at 130 °C under vacuum. The large monoliths are about 100 μm across.

Fig. 2 shows images of **1**, Cu^I^Cl(ttcH_3_), **2** (after NH_3_ treatment), and **3** (after the treatment and drying). It is clear that the original shapes of the crystals were retained during the dehydrochlorination reaction, while the colour turned from clear red to shiny black. Since **2** and **3** are amorphous to X-rays (Fig. S2†), they are monoliths rather than crystals. Elemental analysis revealed that all the chloride was successfully removed from **1**. The composition of **3** was calculated as Cu_1.8_(ttcH_1.2_), *i.e.*, Cu^I^_1.8_(ttc)_0.6_(ttcH_3_)_0.4_ or equivalent, assuming that the linker molecules are intact,^24^ indicating that 44% of the ttc^3–^ ions are removed from **1** and that **3** is richer in copper. **2** has more N and H than **3**, and the composition was found to be (H_3_O)_0.7_(NH_4_)_0.5_[Cu_1.8_(ttc)], assuming that the additional N and H atoms are assignable to NH_4_^+^ cations and H_2_O molecules, as supported by the TGA data (Fig. S3†) and the FTIR data (Fig. 4) discussed below.

### Conductivity measurements

**Fig. 3:**
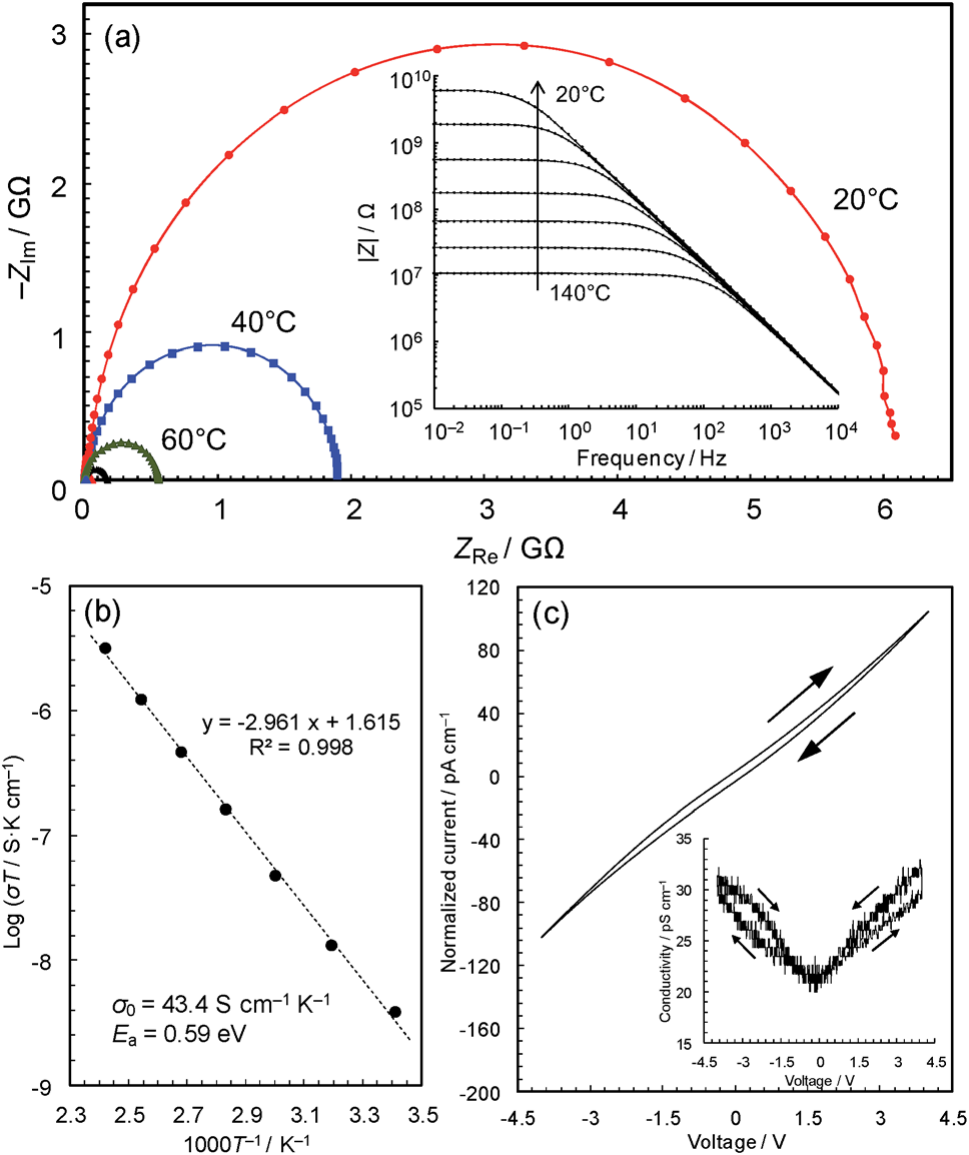
AC impedance analyses. (a) Complex-plane impedance data for a pellet of **3** (red: 20 °C; blue: 40 °C; green: 60 °C; and black: 80 °C). The inset shows log|*Z*| *vs.* log frequency plots. (b) Arrhenius plot of the electronic conductivity. (c) Current–voltage characteristics measured by cyclic voltammetry scanned at 10 mV s^–1^ at 25 °C.

The black colour of **2** and **3** suggests a narrower band gap, which motivated us to measure their electrical conductivities. Fig. 3a shows the temperature dependence of the AC impedance spectra of **3** (details are shown in Fig. S4†). The spectra have single semicircles, indicating that **3** is an electronic conductor (**1** was confirmed to be an insulator using the single-crystal method, Fig. S5 & S6†). The spectra of **3** were analyzed using the Randles equivalent circuit,^23^ and the conductivities were found to be 4.2 × 10^–11^ S cm^–1^ at 20 °C and 7.6 × 10^–9^ S cm^–1^ at 140 °C.

The Arrhenius plot (Fig. 3b) shows linear behaviour from 20 to 140 °C, with an activation energy of 0.59 eV and a pre-exponential factor of 43.4 S cm^–1^ K^–1^. This temperature dependence suggests that conduction proceeds by hopping, or a thermal activation process, rather than a tunnelling mechanism, which is often the case of charge transfer through molecules.^25^ In coordination polymers and/or amorphous materials, electron conduction is likely to follow a localized conduction model such as variable range hopping, where the logarithm of conductivity has a linear relationship with *T* ^–1/(1*+d*)^ (*T* is the absolute temperature and the *d* value represents the dimensionality of conduction network).^7^ We have plotted the temperature-dependence data based on conduction pathways of different dimensionalities (Fig. S7†), although because data could be fitted to all (as is sometimes observed),^26^ the results are inconclusive.

The current–voltage (*I*–*V*) characteristics of **3** (Fig. 3c) essentially shows a linear relationship with a slight inverse S-shaped feature. The conductivity *vs.* voltage plot (Fig. 3c, inset) shows ∼50% increase of conductivity with increasing voltage from 0 to 4 V, pointing to weak non-ohmic behaviour, which is probably due to the presence of non-ohmic contact between the metal electrodes and the material. The DC conductivity of the sample at 0 V, 2.1 × 10^–11^ S cm^–1^, was confirmed to be identical to its AC conductivity. The conductivities are modest compared with reported values of Cu-based coordination polymers in the range of 10^–12^ to 10^4^ S cm^–1^ (Table 1), though the change from a crystalline insulator to an amorphous semiconductor is significant and motivated us to carry out a detailed study of this intriguing material.

**Table 1:** Comparison of known electrically conductive Cu-based coordination polymers

Compound	Conductivity/S cm^–1^	Cu valence	Dimensionality^15^ ^*f*^	Coordinating atoms	Cu–Cu neighbour distance/Å	Remarks^*h*^	Ref.
[Cu(2,5-dimethyl-*N-N*′-dicyanoquinonediimine)_2_]^*a*^	1.0 × 10^4^ ^*b*^	+1.33	I^0^O^3^	N_4_	3.89	Metallic	27
[Cu(4-hydroxythiophenolato)]	1.2 × 10^2^ ^*d*^	+1	I^2^O^0^	S_3_	3.24	—	28
[Cu_2_Br_4_(bis(ethylenedithio) tetrathiafulvalenium)]	2.1 × 10^0^ ^*c*^	+1	I^2^O^0^	SBr_3_	2.77–2.78 (trimer)	*E*_o_ = 0.5 eV, *E*_a_ = 0.12 eV	29
[Cu_6_Br_10_(bis(ethylenedithio) tetrathiafulvalenium)_2_(H_2_O)_2_]	5.1 × 10^–2^ ^*c*^	+1	I^1^O^0^	SBr_3_, Br_4_	2.78–2.96	*E*_a_ = 0.021 eV	29
[Cu_9_(SH)_8_(2-pylidinethiol)_8_](BF_4_)	1.6 × 10^–3^ ^*c*^	+1	I^1^O^0^	S_3_, S_4_	3.39–3.80	Little visible absorption, *E*_a_ = 0.11 eV	30
[Cu_3_I(pylimidine-2-thione)_2_]	7.0 × 10^–4^ ^*c*^	+1	I^2^O^1^	NSI_2_, N_2_S_2_, NSI	2.66–2.78 (trimer)	—	31, 32
Cu[Cu(2,3-pyrazinedithiolato)_2_]	6.0 × 10^–4^	+2	I^0^O^3^	N_4_, S_4_	5.37	Microporous, *E*_a_ = 0.19 eV	9
[Cu(SCN) (pyridine-3,4-dicarbonitrile)_2_]	4.3 × 10^–5^ ^*b*^	+1	I^1^O^0^ ^*d*^	N_3_S	6.09	—	33
[Cu_2_Br(isonicotinate)_2_]	1.2 × 10^–5^ ^*c*^	+1.5	I^0^O^2^	O_2_NBr	2.39 (dimer)	—	26
[Cu_3_Br(pylimidine-2-thione)_2_]	6.0 × 10^–8^ ^*c*^	+1	I^2^O^1^	NSBr_2_, NSBr, N_2_S_2_	2.59–2.75 (trimer)	—	31
Cu[Ni(2,3-pyrazinedithiolato)_2_]	1.0 × 10^–8^ ^*d*^, 1.0 × 10^–4^ ^*d*^ ^,^ ^*e*^	+2	I^0^O^3^	N_4_, S_4_	5.30 (Cu–Ni) 6.83 (Cu–Cu)	Microporous, *E*_a_ = 0.49 eV (*E*_a_ = 0.18 eV)^*e*^ *E*_o_ = 2 eV	34
[Cu_6_I_6_(pyridine-4-thione)_4_]	2.0 × 10^–9^ ^*c*^	+1	I^3^O^0^	S_2_I_2_	3.14–3.89	*E*_o_ = 1.69 eV	35
[Cu_1.8_(C_3_N_3_S_3_H_1.2_)]	4.2 × 10^–11^ ^*b*^	+1	I^*x*^O^3–*x*^ (*x* > 1)^*g*^	S_3_, NS_3_	∼3.2	Amorphous, *E*_o_ = 0.6 eV, *E*_a_ = 0.59 eV	This work
[Cu(SCN) (4-cyanopyridine)_2_]	1.0 × 10^–12^ ^*b*^	+1	I^1^O^0^ ^*d*^	N_3_S	6.16	Temperature independent	33
[CuCl(C_3_N_3_S_3_H_3_)]	—	+1	I^0^O^3^	S_3_Cl	4.4	*E*_o_ = 2.0 eV	This work

^*a*^Organic molecules are anion radicals.

^*b*^Measured using pellets at room temperature.

^*c*^Measured using single crystals at room temperature.

^*d*^Measured using thin films at room temperature.

^*e*^I_2_-doped samples.

^*f*^SCN is regarded as inorganic.

^*g*^Considered on the basis of the composition and the coordination spheres.

^*h*^ *E*_o_ is the optical bandgap and *E*_a_ is the activation energy.

We compare the electrical conductivity of compound **3** with known, conductive Cu-based crystalline coordination polymers in Table 1. There are two major classes: one has mixed valence of Cu ions without inorganic connectivity (*e.g.*, [Cu(2,5-dimethyl-*N-N*′-dicyanoquinonediimine)_2_]); the other has Cu(i) ions with infinite inorganic connectivity (*e.g.*, [Cu(4-hydroxythiophenolato)]). In light of this, characterization of the valance of Cu ions as well as its inorganic connectivity is considered to be a reasonable starting point for characterizing our material. Compound **3** was fully characterized by FTIR spectroscopy, XPS, PDF analysis, UV-vis spectroscopy and ESR spectroscopy.

### Chemical bonding

**Fig. 4:**
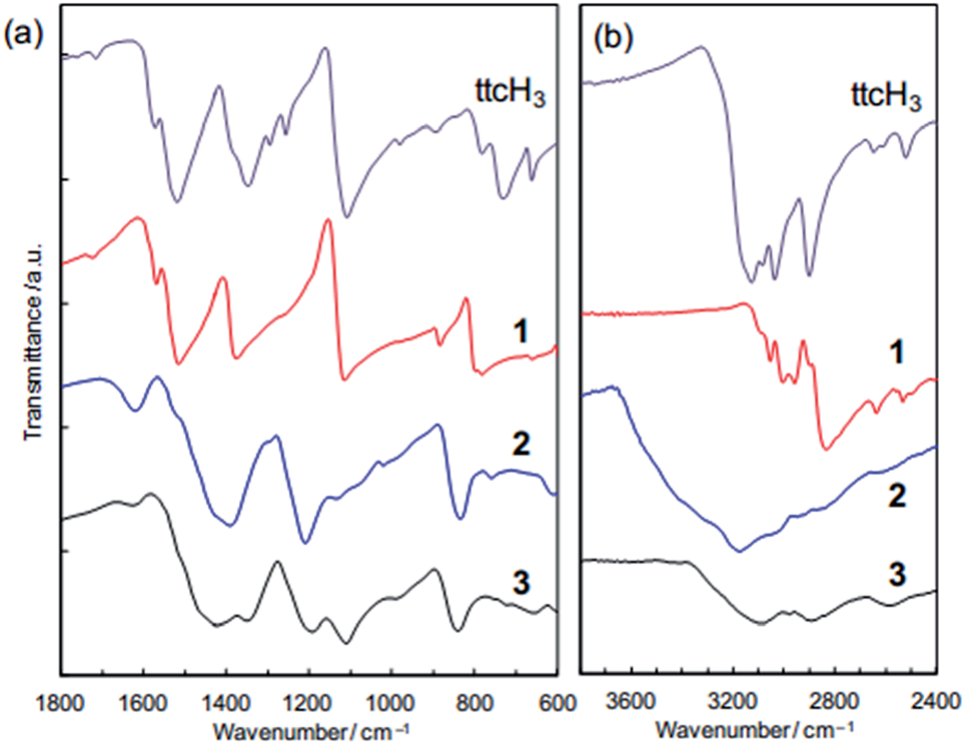
FTIR spectra of **1**, **2** and **3** compared with pure trithiocyanuric acid. (a) 600–1800 cm^–1^. (b) 2400–3800 cm^–1^.

Fig. 4a shows that the FTIR spectra of **1**, **2** and **3**. **1** contains major bands at 1520, 1385, and 1120 cm^–1^ (assignable to S=C bonds), which are consistent with the presence of the neutral ttcH_3_ molecules. This confirms that ttcH_3_ in **1** primarily adopts the non-aromatic, thioketone form.^36^ After the NH_3_ treatment, *i.e.* in **2**, these bands were shifted to 1440, 1200, and 850 cm^–1^ and are assignable to the aromatic, thiol form of the ttcH_3_ molecules.^36^ This is typical for the deprotonated ttc^3–^ anions. Then, after the vacuum drying, *i.e.* in **3**, the spectrum contains peaks assignable to both the thiol form as well as to the original thioketone form. From this we can conclude that **2** contains NH_4_^+^ cations, which require deprotonation of the ttcH_3_ so that it is largely in the thiol form. This is consistent with the elemental analysis. The broad hydrogen-bond bands in **2** (>2600 cm^–1^, Fig. 4b) also point to the presence of NH_3_ and H_2_O, which is confirmed by the TGA (Fig. S3†). The hydrogen-bond bands in **3** shows a small peak assignable to S–H stretching at 2590 cm^–1^ as well as two peaks assignable to N–H stretching at 2900 and 3100 cm^–1^.^37^ FTIR and PDF measurements show that the reaction is completed within 2 days (Fig. S8†).

**Fig. 5:**
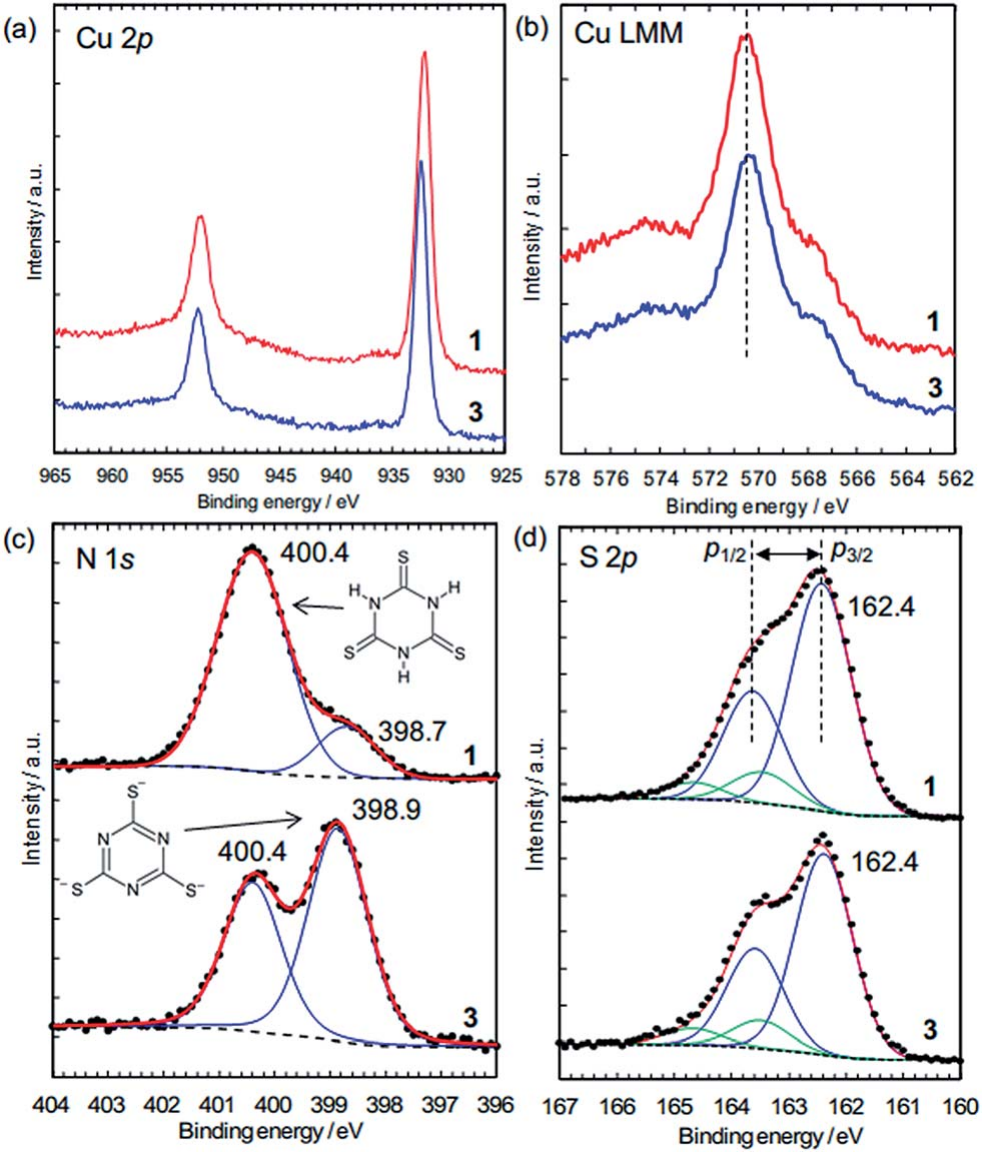
XPS data of compound **1** and compound **3**. (a) Cu 2p spectra. (b) Cu LMM X-ray-excited Auger spectra. (c) N 1s spectra (dots) fitted by two peaks (each: blue lines; total: red lines; and background: dotted lines). The intensity of **3** was magnified by 2.15 times. (d) S 2p spectra (dots) fitted by two sets (blue and green lines) of S 2p_3/2_–S 2p_1/2_ doublet peaks (separation = 1.20 eV; area ratio, 2p_3/2_–2p_1/2_ = 2 : 1). Dotted black lines = background. Red lines = total. The intensity of **3** was magnified by 1.5 times.

The charge states of the Cu ions and trithiocyanurate molecules were investigated by XPS (Fig. 5). The elemental state of Cu could be either Cu(i) or metallic in **1** and **3**, judging by the Cu 2p_3/2_ peak positions (Fig. 5a), which are at 932.2 eV and 932.5 eV in **1** and **3**, respectively. These agree with that in CuCl at 932.4 eV (better than CuCl_2_, 934.4 eV),^38^ and that in Cu(i)–S bonding at 932.3–933.6 eV (note that Cu(ii) ions are probably reduced by thiols).^39^ Furthermore, as in a typical way to distinguish between Cu(i) and Cu(ii), the absence of satellite peaks in the range 940–945 eV (which are typical of Cu(ii))^39^ confirms the elemental state of Cu is Cu(i) or Cu(0). This is distinguished by the X-ray-excited Auger spectra of Cu LMM, which show that the peak positions shown in Fig. 5b (570.5 eV for **1** and 570.4 eV for **3**) are in better agreement with the presence of Cu(i) (570.3 eV) rather than Cu metal (568.1 eV).^40^ Thus, the elemental state of Cu is Cu(i). This is consistent with the dominance of Cu(i) in the conductive Cu-based coordination polymers reported to date (Table 1).

The N 1s XPS peaks located at 400.4 eV (Fig. 5c) are assignable to N–H in the nonaromatic thioketone form of trithiocyanurate, and the peaks around 399 eV are assignable to the aromatic thiol form.^41,42^ Thus, only 16% of nitrogen atoms in **1** are in the thiol form, reflecting the equilibrium between the thiol form and the thioketone form, as observed in the alkali metal trithiocyanurates.^6^ The proportion of this peak increases to 59% in **3**, indicating a negative charge of –1.8/ttc (*i.e.*, (ttc^3–^)_0.6_(ttcH_3_)_0.4_), and this is consistent with the composition determined by elemental analysis. Furthermore, as found by the elemental analysis, there is no chlorine in **3** (Fig. S9†).

The states of the S atoms are similar for **1** and **3** (Fig. 5d), and the peak deconvolution^43^ clarify that there are two sets of S 2p_3/2_–2p_1/2_ doublet peaks (*i.e.*, blue lines and green lines). The binding energies of the main doublet peaks (*e.g.*, 2p_3/2_ peaks at 162.4 eV) (blue lines in Fig. 5d) are consistent with those of pure trithiocyanuric acid molecules (S 2p_3/2_ at 162.3 eV).^41^ Since these peaks are constant upon dechlorination, the anionic charge of the ttc molecules in **3** is considered not to be localized at the S atoms but rather to be in the triazine rings and/or at the Cu(i) ions. On the other hand, the other doublet peaks having 2p_3/2_ peaks at 163.5 eV (green lines in Fig. 5d) are considered to reflect the partial formation of S–H bonds, as 2p_3/2_ peaks of thiol species are in the range of 163.2–163.7 eV.^39^ This is consistent with the FTIR results (Fig. 4b). In addition, these spectra confirm that the ttc molecules are intact, because there are no S–S bonds (which have a 2p_3/2_ peak at 164.4 eV).^41^

### Atomic structure

**Fig. 6:**
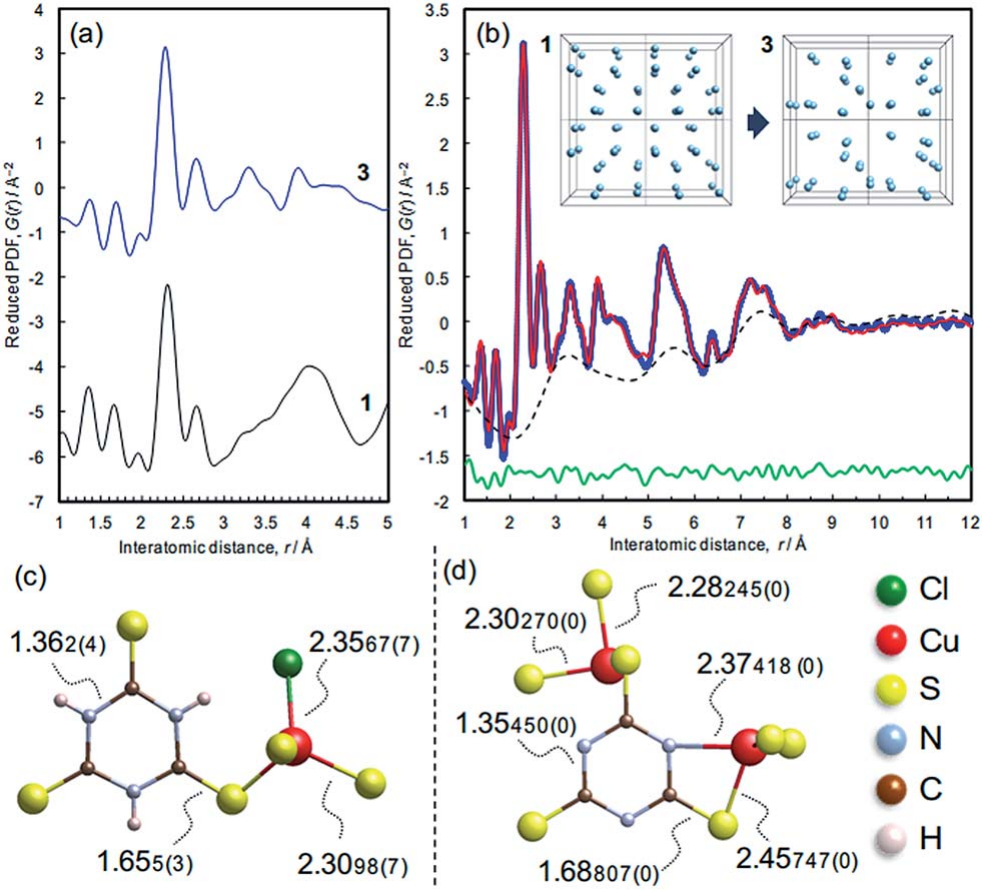
X-ray pair distribution function analysis. (a) Experimental PDF pattern of **3** and simulated pattern of **1**. (b) PDF refinement of the structure of **3**. The model consists of (i) the ttc^3–^ anions coordinating to two Cu atoms as shown in panel d, which generate the sharp peaks, and (ii) the arrangement in space of these units, which generates the broad oscillating background (dotted line). The latter was simulated by moving the coordinates of the Cu sites in **1** to fit the broad oscillation as illustrated in the inset. (c) Local structure and bond lengths of **1**, determined by single crystal X-ray diffraction. (d) Representative local structure and bond lengths in **3**, determined by the PDF refinement.

The atomic structure of **3** was analyzed by the pair distribution function (PDF) method (Fig. 6 and S10†). The experimental PDF of **3** is similar to the simulated PDF of **1** up to 3 Å, indicating that coordination spheres and the linker molecule structure are largely retained during the dechlorination reaction (Fig. 6a). The PDF of **3** (Fig. 6b) shows sharp peaks up to 10 Å, revealing the presence of chemical short-range order. The PDF pattern was analyzed using a model comprising two components as described in the Experimental section (*cf.* Fig. S11†): (i) isolated clusters of linker molecules and Cu coordination spheres, and (ii) disordered arrangements of such clusters within ∼10 Å in order to model the broad oscillation underneath the sharp peaks (Fig. 6b, inset). A reasonable fit between experimental and simulated PDFs is obtained (*R*_w_ = 10.78%),^28^ and the derived local model for the amorphous phase **3** is illustrated in Fig. 6d. Compared with the ttcH_3_ molecules in **1**, the C–S bonds in **3** became longer and the C–N bonds became shorter, corresponding to the formation of the aromatic thiol form, as determined by FTIR and XPS. Note that the broad oscillations cannot be fitted with a model for Cu nanoparticles (Fig. S12†), confirming the absence of Cu(0) as determined by XPS.

In view of the Cu/S ratio of 0.6 and the coordination environments (CuS_3_N and CuS_3_) in **3**, most of the S atoms in **3** must be shared by two Cu ions to form a Cu–S–Cu network, while all the S atoms have a single coordination with Cu ions in **1**. The presence of Cu–S–Cu networks is also suggested by inspection of the cluster arrangement, namely the broad peak in the dotted curve at around 3.2 Å (Fig. 6b) which is assignable to the nearest distance between Cu atoms in the array. Even though this value is not so definitive as the values obtained from the sharp peaks, this distance appears reasonable, considering the nearest Cu–Cu distance in **1** (4.4 Å) and the higher Cu/ttc ratio in **3**. Since direct Cu–Cu interaction is negligible when the distance is >2.8 Å,^14,44^ the conduction is considered to occur through an inorganic CuS_*x*_ network rather than direct metal-to-metal networks. The absence of direct Cu–Cu conduction is typical for Cu-based coordination polymers, where electrons mainly transfer through Cu–S–Cu networks (Table 1 and the references). In other words, the increase of inorganic dimensionality from I^0^O^3^ of **1** can account for the conductivity in **3**, as is often the case of Cu-based coordination polymers (Table 1).

### Outline of the conversion reactions

Shorter reaction times from **1** to **2** led to a product visibly resembling the latter, though retaining diffraction characteristics of the former (Fig. S13†). This indicates that the conversion reaction proceeds from the surface inwards, with an interface between the surface layer of **2** and the core of **1**. When the reaction is completed, the total scattering pattern of **2** is composed mainly of diffuse scattering, and weak peaks not assignable to **1** are observed (Fig. S2a–c†). Upon the removal of the H_2_O and NH_3_ molecules (*i.e.*, formation of **3**), the peaks disappear while the diffuse scattering pattern is retained. This indicates the presence of nanocrystalline phases in **2**, which become disordered when dried.

Reaction at pH 11.4 (which is close to the p*K*_a3_ value of the ttcH_3_ molecules, 11.38),^36^ is essential given that dechlorination is incomplete at lower values and that **1** dissolves at higher pH. We suggest that the ammonia solution at this ‘ideal’ pH enables the partial removal of ttc^3–^ anions, forming pores for the diffusion of Cl^–^ anions and NH_4_^+^ cations. The voids formed by the removal of Cl^–^ anions are found to be too small for their transport (no accessible voids for Cl^–^, which has a radius of 1.81 Å),^45^ by the calculation using the Mercury program for the computationally-prepared structure of Cl-free **1**.^46^ This implies that the ttc^3–^ anions are partially removed and the reaction proceeds from the surface inward. Since the remaining Cu ions need to be coordinated by other ttc^3–^ anions, which coordinate to other Cu ions, it is reasonably considered to form Cu–S–Cu bridges to prevent the ttc^3–^ anions from dissolution. Thus, any further treatment does not change the composition.

Attempts employing lithium hydroxide aqueous solution as an alternative base were unsuccessful, most likely due to prevention of OH^–^ from entering the pores formed by deprotonation of **1**. This is reasonable because the linker molecules in **2** are fully deprotonated and anionic, as found by FTIR and CHN analysis. This in turn indicates the importance of insertion of NH_3_ molecules as found in **2**.

Since the conversion reaction proceeds under the influence of the structural constraints of the initial compound, **1**, and gradually with an interface between the product and the reactant (Fig. S13†), this is considered to be a topochemical reaction during the conversion of **1** to **2**.^47^ The synthesis of amorphous materials *via* topochemical route proceeds step-by-step under the control of the ‘parent’ coordination network, unlike direct precipitation by mixing precursors.^48^ The amorphization reaction used in this work is governed by the difference in chemical potentials of the parent material and the product, which is different from other amorphizations, such as temperature-induced (TIA),^49^ pressure-induced (PIA),^50^ and mechanochemically induced (MIA)^51^ ones, which are governed by heat transfer or pressure. Some of these can proceed *via* a topochemical route if they are ‘chemical reactions’, which are accompanied by bond formation and/or cleavage. The interpretation of reaction mechanisms to form amorphous materials is substantially more difficult than that involved in crystalline material formation, but it is considered important for the synthesis of functional amorphous materials, especially amorphous MOFs.^52^

### Electronic states

**Fig. 7:**
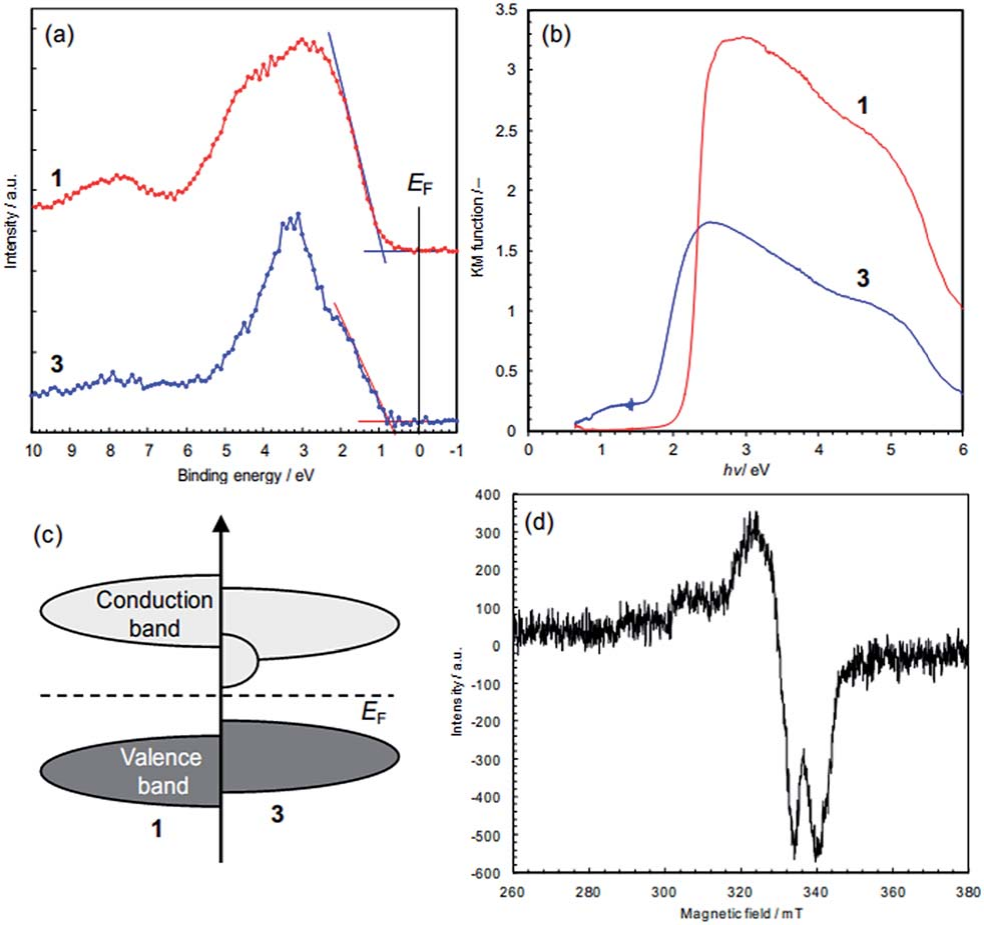
Spectroscopic data of the frontier orbitals in **1** and **3**. (a) XPS data of valence bands. The valence band maxima were obtained by extrapolation of the onset linear parts: 1.0 eV for **1** and 0.8 eV for **3**. (b) Kubelka–Munk functions obtained by diffuse reflectance spectroscopy. (c) Schematic illustration of the positions of the frontier orbitals. (d) X-band ESR spectra for Cu(ii) ions in **3**.

The electronic states of the frontier orbitals were investigated by XPS and diffuse reflectance spectroscopy. The valence band maxima are at about 1.0 eV for **1** and 0.8 eV for **3**, which were estimated by extrapolation of the band edges (Fig. 7a). While **3** does not have inner band states associated with electron donors (Fig. 7a), an additional photo-excitation is observed in **3**, from 0.6 to 1.7 eV (Fig. 7b), showing that **3** has additional unoccupied states just above the Fermi level (Fig. 7c). The optical band gap, *i.e.* 0.6 eV, is close to the activation energy for electrical conduction (0.59 eV). The difference in the electronic states, *i.e.*, colour and conductivity, are attributable to the additional unoccupied states. Our preliminary density functional theory (DFT) calculations using the structures determined by X-ray analysis indicate that the additional unoccupied states in **3** are formed in the S 2p orbitals (Fig. S14†). The removal of chloride ions shifts the Cu d-band upward (Fig. S14a†), that is, charge donation from the S 2p orbitals to the Cu 3d orbitals seems likely. This is consistent with the S 2p XPS data, and the S-to-Cu electron donation is considered to form vacancies in the S 2p orbitals. This influence of dechlorination is observed in **2** as well (Fig. S15†).

In order to probe for electronic defects in **3**, both **1** and **3** were studied by X-band powder ESR spectroscopy (Fig. 7d). These measurements detected unpaired electrons indicative of the presence of Cu(ii), 3d^9^ in **3** (*g* = 2.036), but no paramagnetism was found in **1** (Fig. S16†). Since the signal intensity in **3** is comparable to the Mn^2+^ reference intensity, the concentration is estimated to be <1%, which is too low to be detected by XPS. This 3d^9^ state is considered to represent holes in the valence band, and probably reflects partial charge transfer from Cu to S atoms, though further investigations for the conduction mechanism are needed.

## Conclusions

A crystalline dense MOF insulator, [Cu^I^Cl(ttcH_3_)], has been converted into an amorphous MOF semiconductor, [Cu^I^_1.8_(ttcH_1.2_)], providing another example of the interesting properties that can be found in amorphous MOFs.^52^ The structure and properties of the amorphous phase have been characterized by using a wide range of physical methods. Since our topochemical route can preserve some of the connectivity of the original structure, in this case the CuS_3_ coordination environment, this approach is promising as a post-synthetic treatment for tuning the properties of other MOF materials. Furthermore, the topochemical nature of the reaction underlines once more the flexibility that is exhibited by many dense MOFs, enabling chemical transformations to take place without the complete loss of framework connectivity. Since the conductivity is modest (4.2 × 10^–11^ S cm^–1^ at 20 °C) despite the narrow band gap (optical gap = 0.6 eV; activation energy = 0.59 eV), further detailed analyses and theoretical studies on the mismatch between the apparent conductivity and band gap are needed.
